# 

**Published:** 1851-06

**Authors:** 


### INDEX TO VOLUME SEVENTH.

---

N̆ TICE.—An error occurred in paging the first number of the present volume. The paging was continued from the last volume, instead of commencing anew with this volume. For pages between 761 and 824, the reader is requested to look at the beginning of the volume, viz., the first 64 pages.

Another error in paging is discovered in preparing the Index, viz., from pages 448 to 512, the first figures are 2 and 3, instead of 4 and 5. In preparing the Index the numbers are given as they should have been printed.

☞ *For original articles look for the name of the author as well as the title of the subject.*

*For selected and editorial articles look for the subject alone.*

**A** PAGE.

Abortion brought on by Savin, case of, 796
American Medical Association, Annual Meeting of.................... 809
Am. Medical Association, Circular of Committee of Publication........ 253
Am. Med. Association, Prize Essays.. 306
Am. Med. Association, Transactions of, vol. iv..................... 570
Aztec children, an account of........ 817
Aneurism, Popliteal, cured by compression, by J. R. Wood, M. D..... 105
Appointments, Professorial......... 126
Addresses, Introductory............ 186
Asthma and Œdema Glottidis, differential diagnosis................. 352
Asphyxia from drowning or hanging, treatment of.................... 354
Appetite, extraordinary, and digestion, 355
Arctic Expedition, Gleanings from.. 593
Abscess, Recto-Pharyngeal,......... 675
Association, Am. Med............. 759

**B**

Bladder, Rupture of, Statistics, by Dr. Smith.......................... 822
Buffalo Medical Association, cases reported to....................... 822
Buffalo Medical College............ 823
Burwell, George N., M. D., on Chloroform.......................... 102
Bulwer & Forbes on the Water treatment.......................... 118
Baltimore College of Dental Surgery. 126
Buffalo Medical Association......... 127

PAGE.

Buffalo Medical College, Curators of.. 128
Buffalo Medical College, commencement of session................. 384
Buffalo Medical College, commencement exercises..............575, 639
Barnard, Dr. A., case of Reduction of Dislocated Thigh after Dr. Reid's plan.......................... 279
Bailey, Dr. S. G., case of Reduction of Dislocated Thigh................ 478
Beck's Materia Medica............. 446
Bernard's Lectures on Experimental Physiology...................... 612
Bladder, Treatise on Diseases of, by Prof. Gross...................... 313
Bones, a new method of whitening.. 493
Beck's Infant Therapeutics........ 638
Bloodletting in Surgery, by Ernst G. Pupikofer...................... 641

**C**

Cod Liver Oil, by Dr. Anderson.... 793
Cod Liver Oil in nursing sore mouth. 346
Cod Liver Oil in Consumption, etc... 419
Chloroform administered to Children. By Geo. N. Burwell, M. D........ 102
Chloroform in 9000 cases at Bartholomew's Hospital................. 108
Cæsarean Operation, by M. M. Rogers, M. D.......................... 104
Cataract, on extraction of, by Dr. Williams.......................... 113
Chemistry for Students............ 127
College of Physicians of Philadelphia 128

PAGE.
Chemistry, Elementary, Theoretical and Practical.................. 128
Carcinoma of Stomach, case of, by P. H. Strong, M. D.............. 166
Colegrove, Clinton, M. D., Surgical Notes......................... 206
Conceptions, Extra Uterine, cause of, by S. Hubbard, M. D.............. 215
Clark, Dr., and South Carolina...... 306
Cancer, Lebert on.................. 684
Combustion, Spontaneous.......... 244
Collodion in Skin Diseases......... 248
Concours—Election of M. Nelaton.. 242
Cone, Dr. E. D., case of Monstrosity. 476
Cigars, Medicinal.................. 492
Cragie's Pathological Anatomy..... 509
Croup treated by Tracheotomy...... 553
Carpenter's Elements of Physiology. 628
Cazenave & Schedel's Treatise on Diseases of the Skin................ 638
Cod Liver Oil in Phthisis, by Dr. Walshe.......................... 734
Croup, Remarks on, by Members of the Suffolk Med Society............. 738
Churchill's Midwifery.............. 755

D

Delano, Dr. B. L., on Dislocation of Thigh, etc...................... 780
Dysentery, Treatment of........... 795
Dysentery, Mercury in, by Prof. Spencer............................. 122
Dysentery, Treatment of........... 248
Dysentery, Dr. Parrish on.......... 299
Davis, Dr. N. S., History of Medical Institutions.................... 119
Davis' Introductory Address........ 634
Deaths, Monthly report of, in Buffalo 190, 256, 320, 512, 576, 704
Digestion, Remarks on, by Dr. Lattimore.......................... 272
Draper, Prof., Lecture.............. 338
Dysentery, Epidemic at Girard College........................... 486
Dowler, Dr., and British and Foreign Med. Chir. Review............. 597
Dalton's, Prof., Lectures............ 640
Dickson, Prof., Essays on Life, etc... 660
Damascus, Diseases and Practice of Medicine in...................... 717
Dislocation of Femur into the Obturator Foramen, and Reduction, by Daniel Brainard, M. D............ 723
Dislocation of Femur, Reduction of without Pulleys, by Dr. Luten.... 724

E

Egypt, Medicine in................ 782
Epistaxis, Instrument for arresting... 798
Erie County Medical Society....... 575
Ergot in Parturition, Remarks on.... 682
Education, Preliminary, Editorial... 748

F PAGE.

Fever, Continued, Second Clinical Report on, by Austin Flint, M. D..... 95, 172, 216, 280, 322, 393
Fever, Continued, Supplement to Reports on, by Austin Flint, M. D.... 449
Fever, Continued, Management of, by Austin Flint, M. D.,.......514, 577
Femur, Dislocations of, etc., see thigh.
Flesh and Blood, Extracts of....... 490
Flint, Austin, M. D., Case of Peritonitis............................ 96
Flint Austin, M. D., on Case of Ichthyosis......................... 705
Flint, Austin, M. D., on Cases illustrating the Localization of Valvular Lesions of the Heart................ 709
Fractures, Ununited, Treatment of, by Daniel Brainard, M. D............ 721
Fallopian Tubes, Catheterism of..... 725

G

Gross, Prof, Treatise on Diseases of the Bladder..................... 313
Gregory on Eruptive Fevers........ 316
Gastrotomy, Remarks on............ 261

H

Hamilton, Prof., Trans. of Amussat on Water in Surgery........761, 80, 143
Hamilton, Prof., Letter to.......... 824
Hamilton, Prof., Case of Destruction of Lower Jaw, etc............... 203
Hamilton, Prof., Removal of Lower Jaw.......................... 475
Hæmorrhage, Periodical, from Face.. 798
Hooping Cough, Cochineal in....... 798
Hints on the Profession of Medicine, by Dr. W. M. Wood, U. S. Navy.. 193, 257, 315, 447
House, Dr. J. G., on the Third Stage of Labor....................... 210
Hubbard, Dr. Silas, on Extra-Uterine Conceptions.................... 215
Hubbard, Dr. Silas, on Blending of Twins, etc...................... 546
Homœopathy.................241, 631
Homœopathy, and Prof. Henderson.. 687
Hanging, Death by................ 351
Hunt, Dr. S. B., on Spinal Irritation. 385
Hospital of the Sisters of Charity, Statistical Report.................. 413
Hemorrhoids, Treatment of......... 442
Heart, Rupture of, Case of......... 279
Hospital of the Sisters of Charity.... 507
Hospital of the Sisters of Charity, Second General Report of.......... 541
Heart, Cases illustrating Localization of Valvular Lesions in.......... 709
Homœopathy *vs.* Allopathy......... 719

I

Itch cured in two hours............ 681
Ichthyosis, Case of, with Plate...... 705

J — Page.

Jaw, Lower, Destruction of, etc., case reported by Prof. Hamilton....... 203
Journals, New.................... 446
Jaw, Extirpation of more than half of, by Prof. Hamilton.............. 475
Jenner, Monument to...........497, 576

K

Knee, Articular Inflammation of.... 619

L

Lead, Slow Poisoning by........... 115
Labor, Remarks on Third Stage of, by Dr. J. G. House................. 210
Liver, Cirrhosis of, case reported by Dr. Wilcox..................... 214
Lambert's Physiological Series..... 254
Lattimore, Dr. N. P., Remarks on Digestion....................... 272
Lacteals, Function of.............. 303
Leucorrhea, Nitrate of Silver in..... 347
Liver, Functions of................ 363
Liver, Pus in, Cases illustrative of... 551
Life Insurance of Medical Men...... 683
Lebert on Cancer................. 684

M

Myer, Dr. Albert J., on a New Sign Language for Deaf Mutes.....192, 774
Medical Appointments in New York. 825
Morton, Samuel George, M. D., Obituary........................... 110
Mortuary Reports................ 121
Maguey in Scorbutus.............. 491
Malpractice, Suit for, (Dr. Spencer).. 122
Malignant Diseases, Surgical Operations in........................ 190
Meat, Cooking and Preserving of.... 350
Medical Schools, Primary.......... 380
Molluscum, Two Cases of, by Dr. Ritter........................... 411
Milk from Distilleries, Report on.... 416
Muscular Contraction, Cadaveric Rigidity, French and American Experiments........................ 435
Monstrosity, Case of, by Drs. Cone and Mott...................... 476
Midwifery, Demonstrative, Report of Committee on.................. 498
Microscopist, by Dr. Wythes........ 504
Microscopy, Practical Instruction in, by Prof. Dalton................. 506
Moxon, Dr. E. R., Case of Rupture of Heart......................... 479
Meteorological Register of U. S. Army 510
Medulla Oblongata, Vital spot in.... 616
Monumental Physicians, by the Editor........................... 624
Materia Medica on Stilts, by Wm. Treat, M. D..................... 657
Microscope as a means of Diagnosis,. 688
Microscopical Researches.......... 726

N — Page.

New York State Med. Society, Transactions of...................... 809
New York Medical Schools......... 188
Nashville Journal of Med. and Surgery 192
Notes, Surgical, by Clinton Colegrove, M.D.......................... 206
Northern American Lancet......... 255
New York City University......... 445
New Year....................... 512
New York City Academy of Medicine, Memorial of.................... 573
New York State Med. Society, Transactions of..................... 752
New York Southern Central Association, Transactions of............. 754

O

Ovarian Dropsy, Double, by Prof. Peaslee........................ 820
Officinal Preparations, New......... 191
Ovarian Tumor removed by Catheterism of the Fallopian Tubes, by Dr. Cartwright..................... 237
Os Tincæ, Occlusion of, Case by Dr. Reynale....................... 276
Obstetrical Case, Report of......... 304
Obstetrical Teaching............... 419
Obstruction of Bowels, Treatment of. 422
Obstetrical Auscultation, by Dr. M. M. Rogers......................... 545
Œdematous Laryngitis, treated by Scarifications................... 599
Œdema of Glottis, Symptoms of.... 606
Opium, Large doses of, in the Treatment of Fevers, etc., by Dr. Henry. 667

P

Penis, Amputation of, by Prof. Hamilton.......................... 95
Peritonitis, Case of, with remarks, by Austin Flint, M. D............... 96
Pharmacopœia of United States,..... 124
Phosphate of Lime in Scrofula, etc... 421, 617, 618, 679
Palmer's Patent Leg.............. 432
Patterson, Prof., Death of.......... 447
Puerperal Fever, Prophylaxis of..... 491
Perinœum Prevented by Incision of Vulva......................... 492
Palmer, Prof..................... 510
Pneumonitis, Treatment of, by Editor 567
Pleurisy, Acute, Paracentesis in..... 572
Pupikofer, Dr. Ernst G., Inaugural Thesis on Bloodletting.......... 641
Puerperal Fever Treated with Large Doses of Opium................ 677
Pneumonia, Rules for Bleeding in... 681
Peaslee, Prof., Report of Case of John A. Dobie...................... 727
Phosphate of Lime and Cod Liver Oil in Phthisis.................735, 736
Paracentesis in Pleurisy, by H. I. Bowditch, M. D.................... 740
Pereira's Materia Medica........... 756

**Q** PAGE.

Quackery, Remarks on, by Editor... 689

**R**

Rogers, M. M., M. D., on Cæsarean Operation.......................... 104
Reid, Dr. W. W., on Reduction of Dislocation of Femur,...........129, 561
Regular Irregulars................. 239
Reynale, Dr. W. H., Case of Occlusion of Os Tincæ, etc................. 276
Ramsay, Dr. H. A., and the Am. Med. Association.................307, 443
Rheumatism, Treatment of, by Lemon Juice.......................... 362
Ritter, Dr. H. P., on Molluscum..... 411
Rogers, Dr. J. Kearney, Death of.... 447
Roby on Medical Education,....... 489
Rheumatism, Lemon Juice in....493 672
Rogers, Dr. M., on Obstetrical Auscultation....................... 545

**S**

Stethoscope..................... 12
Strong, Dr. P. H., Case of Carcinoma of the Stomach.................. 166
Storer, D. H., M. D., Address before Mass. Med. Society.............. 184
Sodium, Chloride of, Use of........ 344
Spinal Irritation, Remarks on, by Dr. Hunt.......................... 385
Syphilis, Letters by Ricord.425, 494, 547
Symphysis Pubis, Relaxation of, after Labor.......................... 622
Smith's Operative Surgery......... 630
Skey's Operative Surgery.......... 661
Sodium, Chloride of, a Substitute for Quinia.......................... 680

**T**

Thigh, Dislocation of, by Dr. Delano. 780
Thigh, Easy mode of Reducing, by Dr. Mayr..................... 795

PAGE.

Thigh, Dislocations of............. 353, 442, 723, 724, 756
Tape Worm, Cause and Symptoms of 116
Thigh Dislocation on the Dorsum Ilii, Reducible without Pulleys or any other Mechanical Power, by W. W. Reid, M. D..................... 129
Thigh, Reduction of Dislocation of, after Dr. Reid's plan, by Dr. Barnard 276
Tilt, Dr. E. J., on Diseases of Menstruation, etc.................... 314
Thigh, Reduction of Dislocation of, after Dr. Reid's method, by Dr. Bailey 478
Tracheotomy in Croup............ 553
Testimony, Medical................ 556
Thigh, Reduction of, Communication by Dr. Reid..................... 561
Thomson, Rev. Mr., Sermon........ 639
Treat, Dr. Wm., on Materia Medica.. 657

**U**

Uterine Displacements caused by Dress.......................... 300
Urine, Death from a Dose of........ 469

**V**

Vision, Loss of, etc., by Dr. Stone... 788
Vesico Vaginal Fistula, Cases of by Prof. Haywood.................. 816
Vaginal Discharges in Young Females 243
Veratrum Viride..............558, 623

**W**

Water in Surgery, by A. A. Amussat. Trans. by Prof. Hamilton..761, 80, 143
White, Prof. J. P., Letters from Europe................799, 89, 156, 367
Wood, Dr. W. M., U. S. N., on the Profession of Medicine..........193, 257
Wilcox, Dr. C. H., Case of Cirrhosis of Liver.......................... 214
Williams, Dr. Stephen W., Address.. 694

*New Appointments in the City of New York.*—Dr. Elisha Bartlett, late of the University of the city of New York, has been elected to the chair of Materia Medica in the College of Physicians and Surgeons, vacated by the decease of the late Dr. Beck. Dr. Alfred C. Post, of New York, has been elected to the Chair of Surgery in the University school, vacated by the resignation of Dr. Gross; and Dr. Meredith Clymer, of Philadelphia, has been elected to the Chair of Medicine in the same institution, vacated by the resignation of Dr. Bartlett. Dr. I. M. Carnochan, of New York, has been appointed Prof. of Surgery in the N. Y. Medical College, in the place of Dr. A. L. Cox, resigned. The above we learn from the N. Y. Medical Gazette.

